# Mechanistic in silico explorations of the immunogenic and synergistic effects of radiotherapy and immunotherapy: a critical review

**DOI:** 10.1007/s13246-024-01458-1

**Published:** 2024-07-17

**Authors:** Allison M. Ng, Kelly M. MacKinnon, Alistair A. Cook, Rebecca A. D’Alonzo, Pejman Rowshanfarzad, Anna K. Nowak, Suki Gill, Martin A. Ebert

**Affiliations:** 1 School of Physics, Mathematics and Computing, The University of Western Australia, Crawley, WA Australia; 2 National Centre for Asbestos Related Diseases, The University of Western Australia, Crawley, WA Australia; 3 Institute for Respiratory Health, Institute for Respiratory Health, Perth, WA Australia; 4 School of Biomedical Sciences, The University of Western Australia, Crawley, WA Australia; 5 Centre for Advanced Technologies in Cancer Research (CATCR), Perth, WA Australia; 6 Medical School, The University of Western Australia, Crawley, WA Australia; 7 Department of Radiation Oncology, Sir Charles Gairdner Hospital, Nedlands, WA Australia

**Keywords:** Cancer immunotherapy, Radiotherapy, Mathematical modelling, Treatment response

## Abstract

Immunotherapy is a rapidly evolving field, with many models attempting to describe its impact on the immune system, especially when paired with radiotherapy. Tumor response to this combination involves a complex spatiotemporal dynamic which makes either clinical or pre-clinical in vivo investigation across the resulting extensive solution space extremely difficult. In this review, several in silico models of the interaction between radiotherapy, immunotherapy, and the patient’s immune system are examined. The study included only mathematical models published in English that investigated the effects of radiotherapy on the immune system, or the effect of immuno-radiotherapy with immune checkpoint inhibitors. The findings indicate that treatment efficacy was predicted to improve when both radiotherapy and immunotherapy were administered, compared to radiotherapy or immunotherapy alone. However, the models do not agree on the optimal schedule and fractionation of radiotherapy and immunotherapy. This corresponds to relevant clinical trials, which report an improved treatment efficacy with combination therapy, however, the optimal scheduling varies between clinical trials. This discrepancy between the models can be attributed to the variation in model approach and the specific cancer types modeled, making the determination of the optimum general treatment schedule and model challenging. Further research needs to be conducted with similar data sets to evaluate the best model and treatment schedule for a specific cancer type and stage.

## Introduction

The benefits of radiation therapy (RT) for the treatment of localized cancer include its spatially-localized ablative properties; the induction of localized, catastrophic damage to the tumoral DNA and subsequent cell death. A component of this cell killing is now understood to be a result of the immunogenic nature of radiation-induced cell death [7]. This can induce local bystander and, rarely, remote abscopal effects, where a tumor outside the irradiated region regresses [18]. However, there is a lack of an enduring response in a clinical setting [62].

The addition of immune checkpoint inhibitors (ICIs) capitalizes upon the inflammatory environment induced post-RT. Clinical trials in multiple cancer types have shown improvement in patient outcomes when both immunotherapy (IO) and RT are administered [30, 35, 51, 56]. The mechanisms underlying RT/ICI synergy are poorly understood, and the factors necessary to optimize the treatment even less so. ICI monotherapy itself is a capricious treatment, and the increased complexity of the dual treatment requires further investigation.

There is intense interest in the use of ICIs in combination therapies in clinical and preclinical research. The most common inhibitors studied are antibodies antagonistic to PD-1 (pembrolizumab and nivolumab), PD-L1 (atezolizumab, durvalumab, and avelumab) and CTLA-4 (ipilimumab and tremelimumab).

The PD-1/PD-L1 axis inhibits the growth and activation of T cells [28]. The CTLA-4 axis regulates central immune tolerance. There is a lack of biomarkers that can reliably indicate treatment success for anti-CTLA-4 and PD-1/PD-L1 inhibitors, or predict which patients will suffer severe, potentially life-threatening side effects [16, 46, 47].

To understand the immune effects of RT and the synergy of IO-RT, the optimal treatment schedules and tumor microenvironment (TME) to elicit RT-immune synergy must be found. Exhaustive testing of dose-fractionation protocols and tumor microenvironment factors is not feasible through preclinical or clinical in vivo research alone.

Developments in the understanding of radiobiology and radiation-immune synergy provide the opportunity to characterize the treatment effects on the TME in silico. Robustly designed models, informed by data, can explore the myriad permutations of the system while requiring minimal physical resources, thus offering the opportunity to explore the dose-fractionation landscape to generate hypotheses regarding promising treatment strategies.

This critical review seeks to summarize in silico RT and IO-RT treatment models to consolidate current approaches. No standards are widely followed in the building or reporting of exploratory in silico models; this review seeks to standardize the discussion of non-diagnostic computational models.

## Methods and materials

A systematic review of the literature was performed using the Preferred Reporting Items for Systematic Reviews and Meta-Analyses (PRISMA) approach [48]. Models of IO-RT combinations were the focus of this review, with readers directed to other reviews that concentrated on the action of IO, such as Valentinuzzi and Jeraj’s review of computational models of cancer immunotherapy [59].

### Search strategy

The PubMed, scopus and Web of Science databases were used to search for papers up to August 2023 that included terms related to immunity and/or immunotherapy, radiotherapy, cancer, and computer/mathematical modelling. Due to inconsistency in the language and keywords used to refer to in silico models of cancer therapy, it became necessary to cast a wide net when searching the databases. The conditional searches were optimized for each database and are included in the supplementary details for reproducibility.

Only studies published in English were considered eligible. Conference papers were included in the initial searches, but no relevant conference papers were found to contain models of sufficient quality for discussion in this review.

**Fig. 1:**
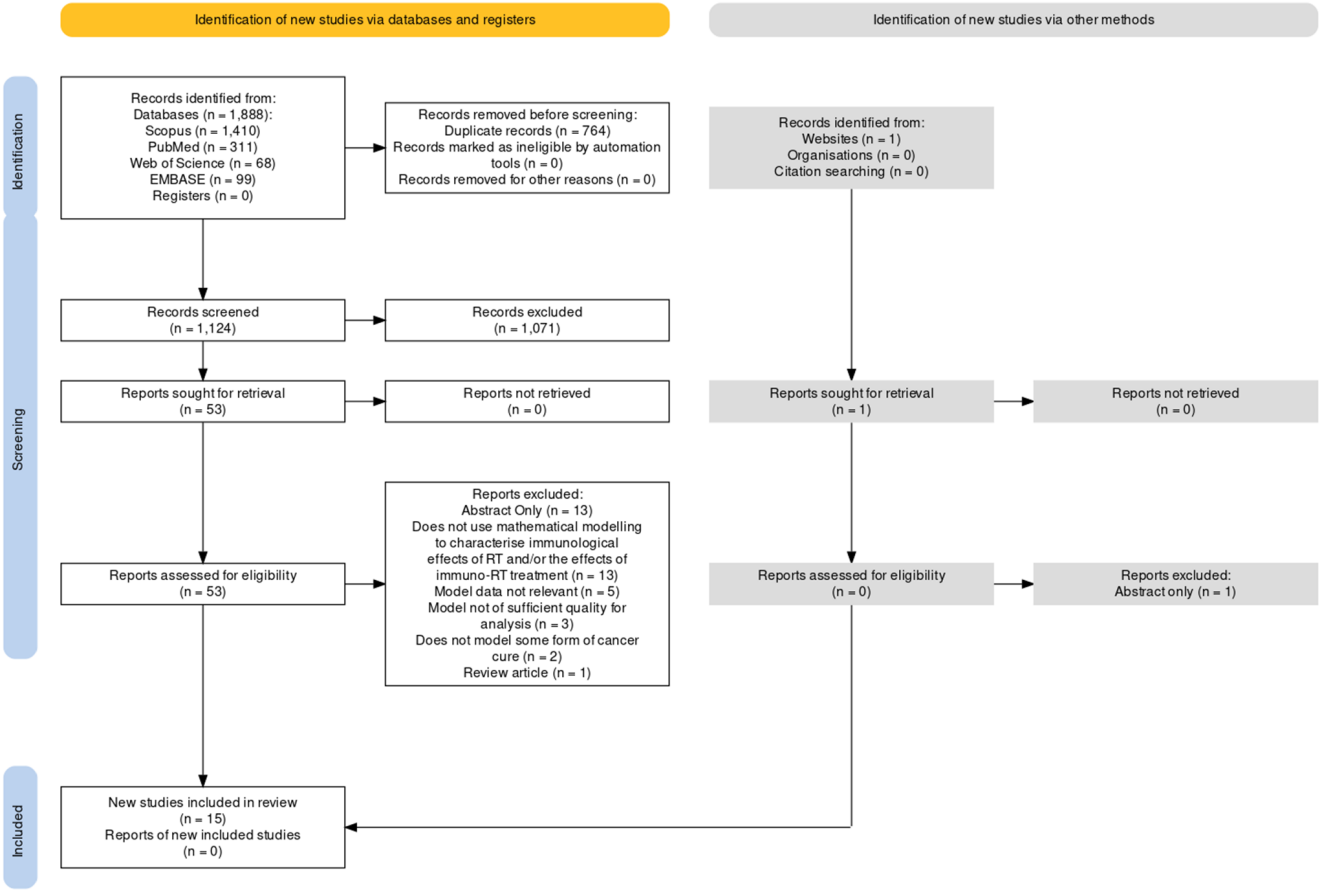
Overview of the study selection process according to the PRISMA guidelines. Figure synthesized using the PRISMA Flow Diagram Shiny app developed by [27]

The breakdown of identified and selected studies according to the PRISMA guidelines is given in Fig. 1. The criteria for inclusion are given in Table 1. Fifteen studies were identified that met the criteria for inclusion.

**Table 1:** Inclusion and exclusion criteria for literature review

Inclusion criteria	Exclusion criteria
• Modeled the effects of combination radiation and ICI therapies on the tumor microenvironment over time • Modeled the effects of radiation therapy on the immune characteristics of the tumor microenvironment over time	• Mathematical model not of sufficient quality for analysis i.e. does not detail model equations, does not relate parameters to biological/physiological function, does not state method of determining coefficient values in article or supplementary material • Does not use mathematical modelling to characterize/explore any of the local immunogenic effects of radiotherapy as part of RT treatment of cancer OR the combination of ICI immunotherapy and radiotherapy OR the immunogenic effects of radiotherapy and how they can/do/may interact with ICI immunotherapy in isolation from the impacts of ANY other intervention • Treatment models only consider the genetic expression • Paper only focuses on remote (abscopal) effects of RT, does not consider local TME effects • Models do not consider changes in the tumor microenvironment over time • Abstract only • Experimental data used in model training/testing does not allow for the exploration of the immunological effects of RT or the interaction of ICI immuno-RT • Mathematical model does not model some metric of tumor cure (e.g. changes in tumor volume, tumor surface area, % of initial tumor size) as an outcome • Review article • Model considers the use of radionuclides in conjunction with a monoclonal antibody or injected radionuclides, not external beam radiotherapy (EBRT)

## Evidence synthesis

### Modeled treatments

The included models seek to understand the interactions between radiotherapy and the immune system in the context of cancer treatment. Some of these models simulate the effects of RT monotherapy on the tumor microenvironment, considering either or both of the immunostimulatory/suppressive effects of the treatment on the TME. The remaining models explore the interactions of radiotherapy with immune checkpoint inhibitors and their effects on the TME. The findings in this section are summarized in Table 2.

#### Subject populations

Most models investigated the effects of RT monotherapy (*n* = 5) or IO-RT treatments (*n* = 10) on either human (*n* = 6) or murine (*n* = 6) subjects, or both (*n* = 2). Patient agnostic models explore potential mechanisms underlying IO-RT treatments rather than focusing on patients with a specific tumor type [3, 54, 55]. The use of murine mathematical models is likely due to the relative availability of murine preclinical data. Most of the available models used external experimental or cancer repository data as part of their modelling process, as opposed to data gathered by the authors for the modelling process. Each model focused on different aspects of the tumor microenvironment after treatment, for instance, immune cell count after RT [57] and kinetic features of immune and tumor cells in murine CT26 tumors [37].

#### Cancer types

Cancer types studied by the models include melanoma, hepatocellular carcinoma, sarcoma, and breast cancer [17, 19, 39, 49, 57, 58].

A few models were cancer agnostic, with the authors choosing to explore mechanisms underlying treatment effects in generality, rather than for a specific cancer type. This means that any agnostic model considers the predictive factors for treatment success to be the same across various cancer types. For a drug to be approved as a cancer agnostic treatment, there should be strong evidence of a molecular aberration–drug match, allowing for extrapolation to diverse tumors, and an understanding of resistance mechanisms to the drug. The effects across different histologies should be homogeneous.

Regarding immune checkpoints, the upregulation or downregulation of these proteins correlates with improved or reduced survival depending on the cancer. For example, the prevalence of BRAF mutations, which promote tumor growth and angiogenesis alone, is not an indicator of treatment success; the BRAF inhibitor vemurafenib was effective in treating melanoma but not colorectal cancer, despite both having a high frequency of BRAF mutations [38]. This means that the bulk of the *in silico* models are of interest in an academic sense but do not apply to predicting the outcomes of individual subjects or treatment groups of specific cancer subtypes.

#### Radiotherapy

All considered models pertained to external beam RT. The RT therapies investigated are most commonly photon EBRT, though one group, Sung et al. [57, 58], models proton radiotherapy [57, 58].

Cho et al.’s research studied spatially fractionated radiotherapy (SFRT), and their results supported Kanagavelu et al.’s findings that conventional RT relied less on immune interaction than SFRT [17, 33]. A single, high dose of SFRT has been reported to induce abscopal and bystander effects, leading to cell death of non-irradiated cells [41]. Additionally, studies have found that SFRT led to a more significant immune response compared to conventional radiotherapy [43]. This is thought to be due to the significantly irradiated tumor cells releasing more antigens, which activate the T cells [43]. Areas of the tumor receiving a lower dose will have a greater degree of vasculature, allowing lymphatic cells to infiltrate the tumor [31]. When the radiation dose was insufficient, the tumor volume exceeded the generalized terminal viable tumor volume before the immune response was activated.

RT not only leads to the death of cancer cells but also causes cell death of immune cells. The cell survival probability for both cancer cells and immune cells is often assumed to follow the linear-quadratic (LQ) model [42]. However, Serre et al.’s model [54] assumes that RT leads to the complete elimination of all immune cells without using the LQ model [54]. Each model considers a different effect on the tumor microenvironment, for example, the short and long-term effects of RT on tissues in the physical, chemical, and biological phases, the interaction of ionized molecules with biological components of the cell [55], and the release of tumor antigens during radiotherapy [3, 19, 44, 54], which leads to the production of immune cells. Sung et al. [58] consider immunosuppressive mechanisms of RT, however, they did not study the enhancement effects of RT which have been observed in other pre-clinical studies [26, 58].

#### Radiotherapy fractionation

The optimal fractionation of RT dose and duration of treatment varies depending on the cancers being treated and the radiosensitivity of organs at risk of receiving the dose during treatment. There is an increased drive to understand the effects of RT total dose, timing of therapy, and fractionation on the TME, especially on the TME’s oxygenation and perfusion.

The models present contrasting results regarding the effect of RT fractionation on treatment efficacy. One model found that hypofractionation led to a higher inactive tumor cell population, which stimulates lymphocytes leading to an expedited recovery period of the immune system [57]. This is consistent with clinical observations that reducing the number of fractions for the same overall dose increases treatment efficacy [32, 60]. However, other models found that long fractionation schemes do not significantly improve the therapeutic success rate compared with shorter schemes [3, 58]. Poleszczuk and Enderling [48] found that the optimal number of fractions increases with the biologically effective dose (BED) delivered [49].

#### Immune checkpoint inhibitors

CTLA-4 and the PD-1 pathway both inhibit immune response: CTLA-4 regulates T cell activation in the early stage of the immune response and PD-1 suppresses T cell activity in the late stages [10]. The majority of the models studying the effect of immunotherapy considered anti-PD-L1 and/or anti-PD-1 drugs, as shown in Table 2. The PD-1/PD-L1 axis plays a significant role in cancer’s evasion of immune defences as the binding of PD-1 to PD-L1 induces apoptosis in the T cells [28]. A few models considered anti-CTLA-4 drugs [36, 49], with some models considering both immunotherapy treatments [17, 19]. Most of the models are drug-agnostic, only considering the generic action of the drug, for example blocking the PD-1 pathway. Byun et al. [13] studied the 10 F.9G2 monoclonal antibody [13], and Poleszczuk and Enderling studied the 9H10 anti-CTLA-4 monoclonal antibody [49]. Kim et al. [35] studied the anti-CTLA-4 drug tremelimumab [36] and Sung et al. [57 studied durvalumab, an anti-PD-L1 immune checkpoint inhibitor [58]. Different immunotherapy drugs have differences in pharmacokinetics and pharmacodynamics, which can affect treatment outcomes depending on the drug used. For instance, avelumab and durvalumab are both PD-L1 inhibitors, however, avelumab has a linear clearance rate over the dose range of 1–20 mg/kg, whilst durvalumab displays a non-linear clearance rate for doses less than 3 mg/kg [15].

#### Exploring the impact of sequencing of immune checkpoint inhibitor and radiotherapy

All the models that considered both RT and IO show that treatment is improved when RT and IO are both administered, as opposed to only administering one or the other.

This indicates an additive effect between RT and IO. However, there is a difference in treatment efficacy when the ordering of the therapies is altered.

Current evidence indicates a difference in IO-RT treatment efficacy depending on whether the ICI or the RT is administered first. The models did not agree on an optimum treatment sequencing; it depends on the doses of ionizing radiation delivered and ICI injected, as well as the type of ICI. For small doses of RT and anti-PD-L1, it is more effective to give anti-PD-L1 after RT, compared to administering IO simultaneously the reverse is true for large doses, as evidenced in Lai and Friedman’s study [39]. This finding aligns with a retrospective analysis of patients with resected melanoma brain metastases, which found that RT followed by IO led to increased patient survival [50]. However, the exploration conducted by Kim et al. indicated that the optimum treatment sequencing depended on the type of IO administered. It was more effective to administer anti-CTLA-4 before RT, but the opposite was true for anti-PD-L1 [36]. This agrees with the experiment conducted by Young et al., where mice with colorectal carcinoma were administered anti-CTLA-4 and RT. Treatment with anti-CTLA-4 before RT increased the proportion of mice with cleared tumors [63].

A few models found that administering RT and ICI concurrently increased the response rate, with the response rate decreasing as the interval between treatments increased [22, 37, 58].

**Table 2:** Key characteristics of experimental design in identified studies

Model	Subject Population	Cancer Type	RT Modeled	RT Fractionation	IO target	IO-RT Sequencing
Alfonso et al. [3]	Human	Cancer Agnostic	Photon EBRT	2 Gy/day 5 days a week^*a*^	N/A	N/A
Alfonso et al. [4]	Human	Non–small cell lung cancer	Photon EBRT	10 Fractions of 2 Gy	N/A	N/A
Butuc et al. [12]	Murine	BCL1 Lymphoma	Photon EBRT	Continuous Radiation	Unspecified	Simultaneous^*b*^
Byun et al. [13]	Murine	Breast Carcinoma	Photon EBRT	12 Gy/4 fractions every 3 days	PD-L1	RT before IO
Cho et al. [17]	Human and Murine	Sarcoma^*c*^ and Breast Carcinoma	SFRT	Varied ^*d*^	PD-L1 and CTLA-4	N/A
Gonzalez-Crespo et al. [14]	Murine	Breast Carcinoma	Unspecified	Varied^*e*^	CTLA-4 and PD-L1	Varied^*f*^
Kim et al. [35]	Human	Hepatocellular Carcinoma	Unspecified	Unspecified	CTLA-4	Varied
Kosinsky et al. [37]	Murine	Colon Carcinoma	Photon EBRT	2 Gy/5 fractions on days 7–11	PD-L1	Varied^*g*^
Lai and Friedman [39]	Human and Murine	Melanoma	Photon EBRT	Varied^*h*^	PD-L1	Varied^*i*^
Montaseri et al. [44]	Murine	Colon Carcinoma	Photon EBRT	1 fraction a day^*j*^	N/A	N/A
Poleszczuk and Enderling [48]	Murine	Breast Carcinoma	Photon EBRT	Varied^*k*^	CTLA-4	RT before IO
Serre et al. [54]	Patient Agnostic	Cancer Agnostic	Photon EBRT	Single Fraction of 8 Gy	CTLA-4, PD-L1, and PD-1	RT concurrent or before IO
Sotolongo-Grau et al. [55]	Human	Cancer Agnostic	Photon EBRT	Varied^*l*^	N/A	N/A
Sung et al. [57]	Human	Hepatocellular Carcinoma	Proton EBRT	15 fractions to a total dose of 58.0 Gy	N/A	N/A
Sung et al. [58]	Human	Hepatocellular Carcinoma	Proton EBRT	8 Gy/3 fractions	PD-L1	Varied

^*a*^The number of fractions was varied between 15, 25 or 35 fractions

^*b*^The system administers a constant amount of radiation in a finite time interval, so both treatments can be considered to be administered simultaneously

^*c*^There was only a single human sarcoma patient used to fit the data. The mice received a xenograft of 67NR breast carcinoma

^*d*^Radiotherapy dose is varied from 3 Gy to 4 Gy, over 5 days

^*e*^RT schedules consisted of: 8 Gy x 3, 5 Gy x 6, and 20 Gy x 1

^*f*^The days on which immunotherapy was administered were varied, with the days that RT was administered being kept constant on days 0, 1, and 2

^*g*^The timing of anti-PD-L1 was varied - administered on either day 5, 7 or 12^*h*^Radiotherapy dose is varied from 3 Gy to 4 Gy, over 5 days^*i*^RT is administered either 1 week before, simultaneously, or 1 week after IO.^*j*^Radiotherapy dose is varied from 2 Gy to 6Gy^*k*^Radiotherapy dose schedule varied - schedules studied are 20 Gy × 1 fraction, 8 Gy × 3, and 6 Gy × 5^*l*^Radiotherapy dose was varied depending on radiosensitivity parameters

### Key characteristics

Broad characteristics have been defined to improve the analysis and streamline the comparison of the available models. Table 3 summarizes the characteristics of each model.

#### Temporal vs. spatiotemporal models

The majority of models assume that tumor characteristics depend solely on time. Characteristics of interest include tumor volume or the total number of tumor cells, immune cell concentration, and concentrations of key proteins such as antigens and cytokines. Poleszczuk and Enderling [48] considered two tumor sites (primary and secondary) and the migration of tumor cells between the two sites [49]. However, spatial dependence is not considered for each site.

There was only one truly spatiotemporal model in the cohort, which investigated the movements of individual chemical or cellular actors describing system evolution in four dimensions [4]. The three-dimensional location of individual cells is a core determinant of tumor outcomes in spatiotemporal models. The immune cells and cancer cells were modeled on two interacting 3D lattices. The migration of cancer cells was modeled by random walks. Lai and Friedman’s model considered some spatial dependence with the movement and diffusion of cells, anti-tumor drugs, and cytokines [39]. However, it is assumed that the cell density is spatially invariant, so the model is not a true spatiotemporal model.

#### Exploratory vs. Ad-Hoc models

The majority of models in the cohort are ad-hoc, data-driven models as they attempt to fit parameter values to patient data. The tumor dynamics for varying parameter values were investigated by exploratory models [12, 44, 55]. Stability analysis and bifurcation diagrams are included in the results obtained from the exploratory models. Most of the models are mathematical as they use ordinary/partial differential equations or sequential functions to model the concentration/volume of cells. All models assumed the forms of the differential equations and sequential functions and did not investigate other forms of the differential equations and sequential functions.

#### Stochasticity to reflect individual fluctuations within the population

Stochasticity is incorporated in some models to replicate the stochastic nature of mechanisms and processes impacting tumor response. Commonly, the movement of cancer and/or T cells was considered to be stochastic, following a random walk [4, 49]. Gonzalez-Crespo et al. [14] used a Markov birth-death stochastic process to model the changes in tumor cell population for a small number of tumor cells (less than 1000 cells) [19]. Alfonso et al. (4) assumed that migration, proliferation and apoptosis of cancer cells are stochastic processes [4]. The immune system dynamics, including migration and suppression of T cells, as well as immune-mediated tumor cell killing, were also modeled as stochastic processes.

#### Use of differential equations or sequential functions

Differential equations are commonly used to model tumor and immune system dynamics and evolution with respect to time. As discussed in the section titled “Temporal vs Spatiotemporal Models”, most models are purely temporal, so ordinary differential equations (ODEs) suffice to describe the system. The cell concentration is discontinuous at times when radiation is applied - these discontinuities are explicitly defined using the LQ model. A couple of papers scale their ODEs to a dimensionless form before attempting to solve them [12, 55]. Notable exceptions to the ODE models use partial differential equations to model the spatial distribution of tumor and immune cells [4, 39].

Some models opt to use sequential functions, which assume changes to the system occur at discrete time steps [17, 54]. The discrete time steps in the models have a length of 1 day. They did not use ODEs to describe dynamics between treatments.

A few models considered both sequential functions and ODEs [36, 57, 58]. The ODEs model the dynamics between RT fractions, and the sequential functions model the dynamics when the RT fraction is administered. The dynamics when the RT fraction is administered can be incorporated into the ODEs with the use of a Dirac-Delta function.

#### Systemic changes characterized by the models

Though all models are fundamentally looking at tumor cure, each model has a distinct approach. The differential equations and sequential functions are the focus of each model and underline the systemic changes the model is investigating as likely contributors towards tumor cure. Common systemic changes of interest include the presence of immune effector and suppressor cells in the TME and the presence of immunosuppressive ligands. All the models considered model the population or density of immune effector and suppressor cells in the TME. A few models considered the presence of cytokines in their models and their interactions with immune cells [4, 39, 55].

#### Common foundation models

Many of the treatment models make use of existing equations describing simpler systems. Some equations are reoccurring in various treatment models. The LQ model is commonly used to predict the fraction of cancer cells surviving a fixed dose. The LQ model has been modified in some models to consider the repair rate and delivery time, varying radiation doses each day, the effect of hypofractionation, and the variation in radiosensitivity between proliferative and quiescent tumor cells [3, 4, 19, 49, 54].

Most models assume that in the absence of RT and IO, tumor growth is logistic [12, 13, 19, 39, 49]. However, a few models assume tumor growth is exponential in the absence of treatment [17, 36, 54, 57]. Natural cell death is often assumed to cause exponential decay in the cell population for both cancer cells and immune cells [49, 57, 58]. However, a few models do not consider natural cell death to occur for cancer cells [13, 19, 57, 58].

Sotolongo-Grau et al. used a Lotka-Volterra model to model the interaction between the tumor cell and lymphocyte population [55]. This model assumes that tumor cells and lymphocytes can grow without bound [55]. Poleszczuk and Enderling [48] did not follow the Lotka-Volterra model for their analysis; only the term representing immunogenic tumor cell death resembles the Lotka-Volterra model [49].

**Table 3:** Key characteristics of models in identified studies

Model	Spatiotemporal Nature	Exploratory vs. Ad-Hoc	Includes Stochasticity	Equation Forms	Tumor Growth Model	Radiation Model	Systemic Changes
Alfonso et al. [3]	Temporal	Ad-Hoc	No	ODEs	Exponential	LQ^*a*^	tumor radius, effector cell population, therapeutic success rate
Alfonso et al. [4]	Spatiotemporal	Ad-Hoc	Yes	PDEs	Probabilistic^*b*^	LQ^*a*^	tumor, effector, and suppressor cell populations, individual radiation immune score
Butuc et al. [12]	Temporal	Exploratory	No	ODEs	Logistic	Exponential decay	tumor and immune effector cell populations
Byun et al. [13]	Temporal	Ad-Hoc	No	ODEs	Logistic	Exponential decay	tumor volume, T cell density, and concentration of PD-L1, PD-1, and PD-1-PD-L1 complex
Cho et al. [17]	Temporal	Ad-Hoc	No	Sequential Functions	Exponential	LQ	Viable and doomed cancer cell volume, primary and secondary immune response, antigen and lymphocyte dynamics
Gonzalez -Crespo et al. [14]	Temporal	Ad-Hoc	Yes	ODEs	Logistic	LQ^*c*^ and LQL	Antigen and drug concentration, tumor and T cell population
Kim et al. [35	Temporal	Ad-Hoc	No	ODEs	Exponential	LQ^*d*^	Irradiated, non-irradiated, and inactivated tumor cells circulating lymphocytes
Kosinsky et al. [37]	Temporal	Ad-Hoc	No	ODEs	Logistic	LQ	tumor volume T cell concentration concentration of PD-L1
Lai and Friedman [39]	Temporal	Ad-Hoc	No	PDEs	Logistic	LQ	tumor cell, macrophage, dendritic cell, and T cell population ligand concentration
Montaseri et al. [44]	Temporal	Exploratory	No	ODEs	Exponential	Exponential decay	Average tumor radius, effector cell concentration, fraction of cells killed by radiation^*e*^
Poleszczuk and Enderling [48]	Temporal	Ad-Hoc	Yes	ODEs	Logistic	LQ	tumor volume, relative T cell density, and survival fraction
Serre et al. [54]	Temporal	Ad-Hoc	No	Sequential Functions	Exponential	LQ	tumor mass, primary and secondary immune response, antigen and lymphocyte dynamics
Sotolongo- Grau et al. [55]	Temporal	Exploratory	No	ODEs	Exponential	N/A	Clonogenic and non-clonogenic tumor and lymphocyte population
Sung et al. [57]	Temporal	Ad-Hoc	No	ODEs	Exponential	LQ^*d*^	Primary, inactivated, and metastatic tumor cells and circulating lymphocytes
Sung et al. [58]	Temporal	Ad-Hoc	No	ODEs	Exponential	LQ^*d*^	Irradiated, non-irradiated, and inactivated tumor cells, circulating lymphocytes

^*a*^An additional parameter scales the radiosensitivity depending on whether the tumor cells are hypoxic or proliferative

^*b*^The probability of mitosis for each time-step of 1 h is quoted. During mitosis, the new cancer cell is placed on a randomly selected free neighbor node

^*c*^Investigated a modification of the quadratic term of the LQ model

^*d*^For survival fraction of circulating lymphocytes, the *D*^2^ dependence was omitted. However, the survival fraction of tumor cells included the *D*^2^ dependence

^*e*^Ligands considered include PD-1, PD-L1, PD-1-PD-L1, IL-12, IL-2, and TGF-*β*.

### Parameters

Both exploratory and ad-hoc models involved fixed parameters which did not vary as the model was run. These fixed parameters were either estimated within a range of feasible values or set to literature values. The ad-hoc data-driven models aimed to fit parameters, such as radiosensitivity and tumor-immune cell interaction parameters, into the experimental data. Some models, particularly those using sequential functions, included stochastic parameters. These stochastic parameters took into account the heterogeneity between different patients. Stochastic parameters also allowed the tumor growth and proliferation to be modeled by a Markov chain in the models involving discrete time steps. The numbers of each type of parameter are summarized in Table 4.

#### Fitted parameters

Parameters such as radiosensitivity and cell survival fraction are fitted to the results of the simulation using differential equations or sequential functions. Methods of parameter fitting include grid search [57], moving mesh method for solving partial differential equations [39], and a least-squares method [19, 49].

Exploratory models do not attempt to fit parameter values to the numerical models [12, 44, 55]. Instead, the results presented are in the form of graphs of results obtained from the simulation [1], for instance, success rate vs. immunostimulation [3], therapeutic diagrams [12] or stability diagrams [55].

#### External parameters

The majority of models rely on data obtained in other literature to give values for external parameters. It was common to use published values for parameters [39, 44, 57]. Two studies relied on experimental data to obtain fixed parameter values [13, 19].

#### Assumed or estimated parameters

The models must estimate parameters, as some parameters have values that can fall within a given range, or they may not have experimentally established values for the particular patient/cancer type. Alfonso et al. (4) assumed a cell cycle length of 35 h, and values for apoptotic and migration probabilities [4]. Sung et al. [57] estimated a baseline cell density, gross target volume, proportion of metastatic tumor cells, and the number of circulating lymphocytes [57].

Montaseri et al. [44] estimated the radiation-induced effector cell recruitment rate and decay rate of immunostimulatory signals from irradiated tumor cells undergoing immunogenic cell death [44]. Lai and Friedman [39] estimated any parameter values that were not taken from the literature [39]. Their parameter estimation procedure was described in the Appendix, and the estimations are based on literature values and steady-state equations.

#### Model complexity

Each model assumes certain properties of cancer and immune cells to simplify it and reduce the number of parameters the model is required to fit. Most models are temporal and assume that the tumor is homogeneous in density. The notable exception is Alfonso et al.’s model (4), which models the TME as a lattice of points that cells can migrate between [4]. Other simplifications include assuming the concentration of the PD-1/PD-L1 complex is time-independent, linearizing the model about the equilibrium point or assuming the tumor to be spherically symmetric [3, 13, 39, 44, 49]. These simplifications lead to results that are different from results obtained when using a more comprehensive model, for instance, overestimation of RT dose required, a bias towards a particular treatment schedule or the model only applies to a certain subset of patients, such as those with a depleted immune system [44, 49, 54].

#### Stochastic parameters

Most models assume a one-size-fits-all approach to cancer treatment, assuming that parameters such as radiosensitivity and growth rate of cells are identical for each simulation. To reflect a population, a model should accommodate heterogeneity of factors that lead to heterogeneity in response. Cells also exhibit variation within the same patient at the genetic, phenotypic, and epigenetic levels [45]. A few models did incorporate population heterogeneity by sampling parameter values from set population distributions. The use of a stochastic model (see Sect. 3.2.3) allows a set of simulation results to be generated from the same initial conditions, improving the reliability of the model and potentially reflecting population heterogeneity that arises from the stochastic nature of underlying response mechanisms. The temporal models incorporate population heterogeneity by sampling various parameter values, such as tumor doubling time, from a given distribution. Distributions used for parameters included uniform [13], log-normal [17, 49, 55], and normal [36, 55, 58]. This incorporation of stochasticity or distribution sampling allows a large sample of virtual patients to be generated, increasing the reliability of the study and establishing confidence intervals for predicted quantities.

**Table 4:** A summary of section “Parameters”

Model	Fitted Parameters	External Parameters	Assumed/Estimated Parameters	Total Parameters
Alfonso et al. (3)	N/A	11	2	13
Alfonso et al. [4]	0	9	5	14
Butuc et al. [12]	N/A	8	6	14
Byun et al.[13]	26	0	0	26
Cho et al. [17]	2	8	9	19
Gonzalez-Crespo et al. [14]	29	9	0	38
Kim et al. [35	1	13	0	14
Kosinsky et al. [37]	8	13	4	25
Lai and Friedman [39]	N/A	36	36	72
Montaseri et al. [44]	N/A	12	2	14
Poleszczuk and Enderling [48]	10	1	0	11
Serre et al. [54]	N/A	0	13	13
Sotolongo-Grau et al.[55]	N/A	0	13	13
Sung et al. [57]	7	4	0	11
Sung et al. [58]	8	7	0	15

### Model analysis

#### System behavior

Most of the models did not conduct bifurcation analysis or analyze the parametric sensitivity and stability of the system. A bifurcation analysis found that changing the radiotherapy action factor on effector immune cells does not significantly alter the bifurcation diagram’s shape - there are still two bifurcation points [12]. However, the bifurcation point occurs for a smaller value of the parameter affected by treatment when the radiotherapy action factor on effector immune cells is larger. A bifurcation analysis was conducted on the equations presented in Montaseri et al.’s [44] paper, however, the effect of RT was not considered for that specific analysis [29, 44, 52].

A couple of models studied included stability analysis [17, 55]. It was found that the tumor will vanish if the immune system’s efficiency over the tumor is greater than 1, and a tumor can escape the immune response if the immune suppression exceeds a certain threshold and the tumor is untreated [17, 55]. The instability of the system increases with the immune suppression effect [17].

Several researchers conducted a sensitivity analysis on their proposed model [13, 19, 39, 57]. It was found that the system is most sensitive to immune-related tumor cell death [19]. However, Sung et al. [57] found that the final tumor and lymphocyte count are most sensitive to its radiosensitivity, with lymphocyte count also strongly depending on its regeneration and decay rate [57].

The correlation between tumor size and parameters differs before and after therapy [13]. For instance, the elimination rate of anti-PD-L1 in tissue and the expression level of PD-L1 on activated T cells had little correlation with tumor size before therapy. After therapy, the parameters were positively linear correlated [13]. The concentration of the sum of PD-1 and PD-L1 was not proportional to tumor size after therapy began, indicating that if tumor size decreases, the correlation between them is deregulated. The synergistic effect of combination therapy was believed to accelerate the dysregulation of this correlation.

### Key parameters affecting outcomes

In the absence of treatment, tumor size, PD-1, and PD-L1 expression were positively and exponentially correlated [13]. The tumor grew exponentially over time. While a single treatment modality (RT or IO) suppressed tumor growth, it did not reduce tumor volume. Notably, RT alone demonstrated better efficacy in suppressing tumor growth compared to IO alone [13]. However, the administering of a combination of RT and IO reduced the tumor volume, highlighting the synergistic interaction between RT and IO [13, 19, 58]. The ad-hoc models accurately reproduced the experimental data [13, 19, 37]. In addition, radiosensitivity was found to be a decreasing function of dose, supporting the use of the linear-quadratic-linear model at high dose fractions [19].

It was found that the probability of immune-mediated tumor elimination (IMTE) depends on the ratio of malignant cell burden/effector immune cell infiltrate and the ratio of effector/suppressor immune cell infiltrate [4]. A high concentration of effector cells led to a greater probability of IMTE. The treatment efficiency is increased when PD-1 is more highly expressed than PD-L1 and when the PD-1 level on T cells is smaller [13]. The total expression level is not positively correlated to tumor size when combination therapy is used. The effect of sequencing IO and RT influenced treatment success [39, 58], as well as the dose-per-fraction administered [19].

### Discussion and further work

A model is promising for future research if it sufficiently describes the dynamics between radiotherapy, the immune system and the tumor cells. The immune system dynamics present additional complexity due to the various immune escape mechanisms of tumor cells [8], radiation killing of immune cells [14], and release of immunosuppressive or immune-stimulating substances from radiation-damaged cells [14].

The models studied in this paper do not have a unified approach to investigating the effects of combining IO and RT. The models tend to focus on very different patients, cancer types, and different treatment schedules. Therefore, it is difficult to quantify the optimal conditions from these models. The different models should be run on data from the same cancer type to evaluate the efficacy of the model.

Cancer agnostic models will have limited utility in predicting the efficacy of treatment schedules for different types of cancer. Predictive factors for treatment success vary across different cancer types [38]. The predictors for treatment success in one type of cancer may differ significantly from the predictors for treatment success in another type. In extreme cases, the model of tumor-immune system dynamics may vary between cancer types. Therefore, these models are of academic interest only. This review included cancer agnostic models to give a more complete overview of the potential models used in analyzing RT-IO combination treatments. Cancer agnostic models can always be tested on a specific type of cancer - Cho et al. [17] extended Serre et al.’s model [54] and tested it on murine breast carcinoma data, and a human sarcoma patient [17, 55].

The models by Sotolongo-Grau et al. [55], Montaseri et al. [44] and Butuc et al. [12] have limited utility for future research [12, 44, 55]. The models are purely exploratory and do not attempt to fit to clinical data. However, these models still have their use in analyzing the stability of the system and calculating a minimum dose for tumor control given certain parameters [12, 44, 55]. Future research could incorporate fitting these models to clinical data to evaluate their utility for clinical research.

Models that utilize sequential functions are limited compared to models that utilize differential equations. The use of sequential functions means that the tumor and lymphocyte populations can only be modeled for discrete-time values [17, 54]. In the case of Serre et al.’s and Cho et al.’s model, the discrete-time step was 1 day [17, 54]. Therefore, if RT or IO were administered on consecutive days, the dynamics of the tumor microenvironment between treatment fractions would be ignored. This limitation can be overcome by converting the sequential functions to differential equations.

The efficacy of radiotherapy and immunotherapy varies across patients [53, 61]. One of the factors influencing differences in treatment response is differences in the tumor microenvironment, which corresponds to different model parameter values for each patient [53]. Therefore, the ideal model will incorporate stochasticity to account for variations in the population. The model will be more reliable as it will be more likely to apply to the population as opposed to a specific individual with specific parameter values. In addition, tumor cell proliferation and death are naturally stochastic processes. The LQ model gives a probability of cell killing and can incorporate stochastic parameters [42].

Cancerous tumors constantly evolve after their growth, resulting in a greater degree of spatial heterogeneity [64]. An example of this spatial heterogeneity is well-vascularized areas of the tumor appearing adjacent to hypoxic areas of the tumor [2]. The most comprehensive models will accommodate spatial heterogeneity and the evolution of the tumor microenvironment in time. However, most of the models studied do not model spatial heterogeneity - the only models that consider the spatial dimension are Alfonso et al.’s model [4] and Lai and Friedman’s model [4, 39]. Lai and Friedman’s model is not truly spatiotemporal as it assumes a homogeneous cell density throughout the tumor [39].

The models developed must be tested to ensure they are a good fit for experimental data. The models reviewed are capable of predicting results from experimental data. In Alfonso et al.’s model (2021), the quality of predictions was dependent on the Radiosensitivity Index and Individual Radiation Immune Score (iRIS), where patients with low iRIS scores had the best predictions [4]. The iRIS metric developed by Alfonso et al. (2021), quantifies the likelihood that radiation will induce an increased immune eradication of tumor cells [4]. A lower iRIS value represents a more radiation-responsive tumor microenvironment. iRIS scores were accurately predicted given the properties of different cancers, for example, radiosensitivity. The p-value for the similarity between the median iRIS of lung adenocarcinomas and lung squamous confirms the expectation that the iRIS score should differ between tumors.

Two of the models were capable of simulating primary and secondary tumor volume and tumor rejection probability, which matched the experimental values relatively closely [49, 54]. Butuc et al.’s model [12] explains tumor dormancy and radioresistance. The model characterizes two regions qualitatively: a region where the tumor remains dormant with a low and constant cell count, and another exhibiting radioresistance [12]. Tumor dormancy was achieved for doses greater than 0.5 Gy.

Alfonso et al’s model (2021) is most likely to produce predictions that align with observations in clinical trials. The use of a stochastic spatiotemporal model is more effective at capturing the tumor microenvironment response to radiotherapy. However, the model is lacking as it does not model the effect of immunotherapy on the tumor microenvironment. The model can always be extended to consider immunotherapy - Sung et al. [58] extended their previous model (2020) to incorporate immunotherapy [57, 58]. Spatiotemporal models of immune checkpoint inhibitors and the tumor microenvironment have been developed; however, these were not included in this review as they did not incorporate the effect of radiotherapy [5, 11].

There are barriers to using these mechanistic models for clinical research [40]. *In silico* models are often simplified to reduce the computational complexity of the problem; this can give inaccurate results when the model is applied to a different type of cancer [9]. The quality of the model depends strongly on the quality of the data used to fit the model parameters. A small dataset may result in overfitting the data points when determining the parameter values, particularly those with many parameters [9]. This can introduce bias towards a particular patient demographic if the sample is not representative of the population [40]. In addition, the lack of availability of metastatic cancer data means that the models’ ability to predict cancer metastasis accurately is limited [40]. This is a significant limitation as 2 in 3 cancer deaths arise from metastatic tumors [21]. Therefore, most mechanistic models are of academic interest only and are not commonly used in clinical research [9].

For a mechanistic model to be implemented in clinical practice, the values of its parameters must either be directly measured or fitted to experimental data. In vitro experiments can be used to measure parameters such as tumor cell proliferation, DNA damage, and apoptosis of tumor cells [34]. The surviving fraction of cells after irradiation can be used to determine the radiosensitivity parameters of the tumor [23]. The model’s performance can be evaluated by comparing the fitted parameter values to those obtained in in vitro experiments. Two of the authors attempted to measure parameters, such as PD-L1 expression and immune cell infiltrate composition proportions [4, 37]. Others have fitted their models to published tumor data, such as the data obtained by Dewan et al., to obtain parameter values [20].

Mechanistic models such as those discussed in this review paper provide insight into treatment mechanisms and can be utilized to train machine learning (ML) models [6, 25]. Utilizing mechanistic models to train ML models reduces overfitting and allows ML models to suggest new treatment plans or the effect of a new treatment plan where there is no relevant past data [25]. Artificial intelligence (AI) and ML have demonstrated success in predicting the immunotherapy effects on lung cancer [24]. ML offers several advantages, including it is non-invasive and the objectivity PD-L1 level determinations, which are not subject to observer bias. The AI model is trained based on imaging radiomic features, pathological slice images, mRNAs, miRNAs, and Methylated CpG sites [24]. As data availability increases, ML models trained by mechanistic models will likely be utilized to predict treatment outcomes. In addition, AI can be utilized to approximate complex mechanistic models, particularly when rapid solution or a large number of simulations are required [40].

## Conclusion

The development of immunotherapy as a potential cancer treatment has led to research investigating the effects of combining immunotherapy with established radiotherapy. Many of the models considered were tested against experimental data, except for a few exploratory models.

Differential equations and sequential functions were used to model the interactions of the anti-PD-L1 checkpoint and RT. A few common foundation models, such as the linear-quadratic model and logistic growth, were found across the models. Simplifications such as only considering time dependence and assuming a spherically symmetric tumor were made to streamline the solution. In addition, the parameter values were often considered to be fixed, as opposed to exhibiting stochasticity to account for fluctuations between individuals.

The results of the simulations showed that the most effective treatment plans are when RT and IO are administered concurrently, due to the synergy between the treatments. There are some inconsistencies between the model results as the models focus on different cancer types. The fall-off in tumor response rate is more rapid when RT is started after IO [58]. On the other hand, another model found there was not a significant difference between administering RT first compared to IO [39]. Additionally, there were contrasting results between long fractionation schemes and short fractionation schemes [3, 19, 57].

To evaluate which model is optimal, the models must be run on similar cancer types. When the optimal model is determined, an optimum treatment schedule can be determined for the different types of cancer.

## Appendix A search conditionals

Note that the literature search was kept broad as the known papers had wide diversity in the language used to describe this form of in silico research. Preference was given to initially identifying a larger pool of potential studies and subsequently refining with attention to the inclusion and exclusion criteria. Excluding only items that were articles removed some of the known studies.

A validation set was used: Byun, Kosinsky, Serre, Alfonso. As long as these papers came up across the databases, the search was considered accurate.

Based upon the above keywords, the conditionals for the three main databases are:

### Scopus

Searches both American and British spellings automatically Uses { } for the exact phrase, “” for the approximate phrase.

#### Test one

(TITLE-ABS-KEY (radiat* OR irradiat* OR radiothera* OR radioimmuno*) AND TITLE-ABS-KEY (immun*) AND TITLE-ABS-KEY (Biological model OR Mathematical model OR Mathematical bioscience OR In silico OR (predictive W/2 model)) AND TITLE-ABS-KEY (cancer OR tumor OR neoplasm OR carcinoma))

#### FINAL: (994) did not include Alfonso paper

(TITLE-ABS-KEY (radiat* OR irradiat* OR radiothera* OR radioimmuno*) AND TITLE-ABS-KEY (immun*) AND TITLE-ABS-KEY (Biological model OR Mathematical model OR Mathematical bioscience OR In silico) AND TITLE-ABS-KEY (cancer OR tumor OR neoplasm OR carcinoma))

#### FINAL: (1410) exclude all items that are not articles

(TITLE-ABS-KEY (radiat* OR irradiat* OR radiothera* OR radioimmuno*) AND TITLE-ABS-KEY (immun*) AND TITLE-ABS-KEY (Biological model OR Mathematical model OR Mathematical bioscience OR In silico) AND TITLE-ABS-KEY (cancer OR tumor OR neoplasm OR carcinoma)) AND (EXCLUDE DOCTYPE, “re”) OR EXCLUDE (DOCTYPE, “cp”) OR EXCLUDE (DOCTYPE, “no”) OR EXCLUDE (DOCTYPE, “ed”) OR EXCLUDE (DOCTYPE, “sh”) OR EXCLUDE (DOCTYPE, “ch”) OR EXCLUDE (DOCTYPE, “le”).

### PubMed

Searches both American and British spellings automatically Uses{ } for the exact phrase, “” for the approximate phrase

#### Test 1: (101) does not have Alfonso, Serre, or Byun

(((radiat*[Title/Abstract] OR irradiat*[Title/Abstract] OR radiothera*[Title/Abstract] OR radioimmuno*[Title/Abstract]) AND (immun*[Title/Abstract])) AND (Biological model[Title/Abstract] OR Mathematical model[Title/Abstract] OR Mathematical bioscience[Title/Abstract] OR In silico[Title/Abstract])) AND (cancer[Title/Abstract] OR tumor[Title/Abstract] OR neoplasm[Title/Abstract] OR carcinoma[Title/Abstract])

#### FINAL: (315) does not have Alfonso or Serre (does have Byun)

(((radiat* OR irradiat* OR radiothera* OR radioimmuno*) AND (immun*)) AND (“Biological model” OR “Mathematical model” OR “Mathematical bioscience” OR “In silico”)) AND (cancer OR tumor OR neoplasm OR carcinoma).

#### FINAL: (311) included only full articles, could not exclude review articles etc. Looking at only title and abstract seemed too few results

(((radiat* OR irradiat* OR radiothera* OR radioimmuno*) AND (immun*)) AND (“Biological model” OR “Mathematical model” OR “Mathematical bioscience” OR “In silico”)) AND (cancer OR tumor OR neoplasm OR carcinoma).

### Web of Science

Searches both American and British spellings automatically Uses{ } for the exact phrase, “” for the approximate phrase

#### FINAL: (72)

(TI=(radiat* OR irradiat* OR radiothera* OR radioimmuno*) OR AB=(radiat* OR irradiat* OR radiothera* OR radioimmuno*) OR KP=(radiat* OR irradiat* OR radiothera* OR radioimmuno*)) AND (TI=(immun*) OR AB=(immun*) AND KP=(immun*)) AND (TI=(“Biological model” OR “Mathematical model” OR “Mathematical bioscience” OR “In silico”) OR AB = (“Biological model” OR “Mathematical model” OR “Mathematical bioscience” OR “In silico”) OR KP = (“Biological model” OR “Mathematical model” OR “Mathematical bioscience” OR “In silico”)) AND (TI=(cancer OR tumor OR neoplasm OR carcinoma) OR AB=(cancer OR tumor OR neoplasm OR carcinoma) OR KP=(cancer OR tumor OR neoplasm OR carcinoma)).

#### FINAL: (68) exclude all items that are not articles

(TI=(radiat* OR irradiat* OR radiothera* OR radioimmuno*) OR AB=(radiat* OR irradiat* OR radiothera* OR radioimmuno*) OR KP=(radiat* OR irradiat* OR radiothera* OR radioimmuno*)) AND (TI=(immun*) OR AB=(immun*) AND KP=(immun*)) AND (TI=(“Biological model” OR “Mathematical model” OR “Mathematical bioscience” OR “In silico”) OR AB = (“Biological model” OR “Mathematical model” OR “Mathematical bioscience” OR “In silico”) OR KP = (“Biological model” OR “Mathematical model” OR “Mathematical bioscience” OR “In silico”)) AND (TI=(cancer OR tumor OR neoplasm OR carcinoma) OR AB=(cancer OR tumor OR neoplasm OR carcinoma) OR KP=(cancer OR tumor OR neoplasm OR carcinoma)).

### EMBASE

Searches both American and British spellings automatically Uses{ } for the exact phrase, “” for the approximate phrase

#### Test 1: (594)

((radiat* or irradiat* or radiothera* or radioimmuno*) and immun* and (“Biological model” or “Mathematical model” or “Mathematical bioscience” or “In silico”) and (cancer or tumor or neoplasm or carcinoma)).mp. [mp = title, abstract, heading word, drug trade name, original title, device manufacturer, drug manufacturer, device trade name, keyword heading word, floating subheading word, candidate term word]

#### Test 2: (211) Byun, Kosinsky, Serre included

((radiat* or irradiat* or radiothera* or radioimmuno*) and immun* and (“Biological model” or “Mathematical model” or “Mathematical bioscience” or “In silico”) and (cancer or tumor or neoplasm or carcinoma)).ab, kf, kw, ot, ti.

#### FINAL: (214), added in British spelling of ‘tumour’

((radiat* or irradiat* or radiothera* or radioimmuno*) and immun* and (“Biological model” or “Mathematical model” or “Mathematical bioscience” or “In silico”) and (cancer or tumor or tumour or neoplasm or carcinoma)).ab, kf, kw, ot, ti.

#### FINAL: (99) exclude all items that are not articles

Test 2. and “Article” [Publication Type].
